# Liver Graft Revascularization by Donor Portal Vein Arterialization Following “No Touch” Donor Hepatectomy

**DOI:** 10.1155/1988/97019

**Published:** 1988

**Authors:** A. G. R. Sheil, J. F. Thompson, M. S. Stephen, J. C. Graham, A. A. Eyers, M. Bookallil, M. Kalpokas, G. W. McCaughan, S. F. A. Dorney, H. B. N. Ekberg, D. Mears, G. E. Kelly, K. Woodman

**Affiliations:** 1 Australian National Liver Transplant Unit Royal Prince Alfred Hospital Children's Hospital Camperdown Australia; 2 Department of Surgery University of Sydney NSW 2006 Australia

## Abstract

Unsatisfactory immediate function of the transplanted liver together with technical complications contribute to a persisting early mortality for hepatic transplantation in the 20% range. We report our initial clinical experience with methods, one not previously used clinically, that resulted in uniformly well-functioning liver grafts in 11 patients and contributed to a satisfactory success rate for the procedure. Donors were heart-beating. During the donor operation all manipulations of the liver were avoided until after cold preservation, achieved by external cooling at the same time as circulatory interruption, donor exsanguination and perfusion of the liver with cold oxygenated fluid of “extracellular̵ type. The organs were then gently dissected. At transplantation the livers were revascularized with arterial blood shunted from the recipient iliac artery to the graft portal vein after completion of the suprahepatic IVC anastomosis. The infrahepatic IVCs and hepatic arteries were then joined, the iliac artery shunts discontinued and the portal veins joined. Total ischaemic intervals for the allografts were 3½–8 (average 5). Anhepatic intervals were 1–2¼ (average 2). The arterio-portal shunts were operating for 18–85 (mean 46) min. Blood loss and haemodynamic, acid-base and electrolyte abnormalities at revascularization were minimal. All grafts secreted bile immediately and all parameters reflected continuing improvement of liver function thereafter. Nine patients (82%) are alive between 4 and 18 (mean 11) months after transplantation. We conclude that these methods offer effective avoidance of serious organ damage during donor hepatectomy and preservation, reduced allograft ischaemic interval and reduced recipient anhepatic time. They result in avoidance of blood loss at the time of revascularization, together with minimal haemodynamic, acid-base or biochemical changes. In addition, they allow the surgeon to perform and test all anastomoses without time constraints, provide the capability to deal with unexpected complications, and assure good early graft function.

## INTRODUCTION

The results of liver transplantation have improved remarkably in recent years. Nevertheless, perioperative mortality remains in the range 6–30% even in the hands of experienced groups[1–4]. A feared and often fatal complication is unsatisfactory immediate function of the transplanted liver. The incidence of this complication varies considerably from centre to centre and, in individual centres, from time to time. Shaw *et al.*[5], in analyzing the Pittsburgh experience since the introduction of cyclosporin, reported a primary non-function rate of 4.3%. Kormos *et al.*[6] recording the results of multiple organ procurement in the Pittsburgh region, reported that five (13%) of 49 livers failed to function and a further two had late function. Scharschmidt[7] analyzed the causes of death that occured in 51% of 540 patients who received liver grafts in four major centres (Pittsburgh, Cambridge/King's, Groningen, Hanover) since 1980, and found that hepatic failure, haemorrhage and operative/technical factors contributed 57% of the failures that occured in less than three months from operation, and this resulted in the deaths of 31% of patients.

In our initial clinical experience we have employed a method of graft revascularization differing from that in general use. This method was researched and published by us many years ago[8], but it has not been applied clinically until our own programme started at the beginning of 1986. The method has been used in 11 patients who received liver grafts at least three months ago. There was good immediate function in all grafts. Nine of these patients (82%) are now surviving between 4 and 18 months (mean 11 months) since transplantation.

## MATERIALS AND METHODS

### *Donors*

Donors were individuals less than 50 years of age who had been certified as brain dead. Donor hepatectomy was always part of multiple organ procurement. Skilled anaesthetic support was provided during the operation for maintenance of haemodynamic stability by means of infusions of electrolytes, colloid solutions and blood. Before interruption of the circulation, herapin (30 000 IU if adult and 15 000 IU if paediatric) and methylprednisolene (1g adult; 500 mg paediatric) were administered.

The methods used for donor hepatectomy were modified from those described by others[9,11]. However, we avoid all manipulations of the abdominal organs to be removed (usually the liver and kidneys) until the organs are cold-perfused. We have dubbed this the "no touch" technique. Briefly, a full-length incision from the suprasternal notch to the pubis is made. The common bile duct, gastroduodenal, splenic and left gastric arteries are divided. The superior mesenteric and splenic veins are isolated behind the head of the pancreas. The origin of the superior mesenteric artery is encircled. The infrarenal aorta is dissected free and cannulated for perfusion purposes, with ligation of the inferior mesenteric artery. After heparinization, the superior mesenteric artery, splenic vein and superior mesenteric vein ligated and a cannula is inserted into the portal vein through the superior mesenteric vein. Sponges soaked in saline ice slush solution are placed over the liver and both kidneys. The abdominal cavity is filled with saline ice slush solution. Cold portal perfusion with an oxygenated human albumin solution that has electrolyte concentrations similar to that of extracellular fluid (Table 1) is begun and, if the heart is for transplatation, the cardiac team moves at once to remove the heart. At this time, the aorta is clamped in the lower chest, and organ perfusion via the cannulated abdominal aorta begins. The inferior vena cava is allowed to drain freely into the chest where it has been divided from the heart. If the heart is not to be removed, the right atrium is incised to allow free venous decomposition. Blood and perfusion fluid are removed from the chest cavities by suction. A total of approximately 100 ml of

**Table 1** Initial liver perfusion fluid: concentrations of electrolytes and other constituents.

| *Consituent* | *Concentration* |
|---|---|
| Sodium | 145 mM/1 |
| Potassium | 2.7 mM/1 |
| Chloride | 120 mM/1 |
| Glucose | 5 mM/l |
| Calcium | 2 mM/l |
| Magnesium | 1.3 mM/l |
| Albumin | 45 g/l |
| Mannitol | 1 g/l |
| Heparin | 500 IU/l |
| Ampicillin | 1 g/l |
| Hydrocortisone | 200 mg/1 |

[a] pH 7.4 IU
[b] $pO_2$ 312 ± 74 mmHg
[c] Osmolality 320 mOsm/l
[d] One-litre amounts are made up in 2-litre bags, which are distended with sterile oxygen.
[e] Subsequent perfusion fluid: Red blood cells cross-matched against donor and recipient are added to the perfusion fluid to give a haematocrit of 13% for cold and warm perfusion immediately prior to revascularization.

perfusion fluid per kilogram body weight is used. After 5–10 min exsanguination and perfusion are complete. The cold saline solution is removed from the abdominal cavity by suction. All mesenteric attachments of the bowel are divided and the bowel is removed from the abdominal cavity, fully exposing the retroperitoneum. The liver and kidneys are then carefully dissected while being protected at all times with cold sponges.
The liver is transported immersed in the same perfusion fluid as used for perfusion.

### *Recipients*

Recipients were four children aged 2–17 (mean (*m*) 12) years and seven adults aged 20–50 (*m* 37) years. Causes of hepatic failure were primary sclerosing cholangitis [4 cases], secondary biliary cirrhosis [2], alpha-1 antitrypsin deficiency [2], primary biliary cirrhosis [1], tyrosinaemia [1], and Wilson's disease [1]. Four (36%) recipients fell into the high-risk group defined by Shaw *et al.*[4] because of portal vein thrombosis after a previous meso-caval shunt [1], previous porto-caval shunt [1], multiple biliary operations with intra-duct stones and pancreatic abscess [1], and advanced cahexia with persistent variceal haemorrhage and encephalopathy [1].

The methods used for implantation of the livers were again modelled upon those of the pioneers of liver transplantation, including the use in adults of veno-venous by-pass methods without the administration of heparin during the anhepatic interval[5]. However, at the same time as the left groin was prepared for veno-venous by-pass, the left superficial femoral artery was isolated for subsequent insertion of an iliac arterial shunt. When the recipient liver had been prepared for removal, veno-venous by-pass was established and a clamped arterial cannula filled with heparinized saline was inserted via the left superficial femoral artery into the left external iliac artery. The recipient liver was then excised. After completion of the first anastomosis [the suprahepatic inferior vena cava (SHIVC) at the level of the diaphragm], the liver was perfused with cold oxygenated perfusate now containing red blood cells crossmatched against both donor and recipient to a haematocrit of 13%. The caval anastomosis, hilar region and retrophepatic caval area were inserted for leakage, and any defects were secured. Effluent was allowed to flow freely from the unclamped infrahepatic IVC. Samples were taken for analysis at the beginning of, during and at the completion of perfusion.

In the first three patients the method of revascularization by means of an arterio-portal shunt was tested only after the infrahepatic IVC and arterial anastomoses were completed, but not released. The arterial shunts into the graft portal veins were opened as the sole method of revascularization for 10, 15 and 20 min during the first, second and third operations, respectively. When it was found to be safe, the following procedure was standard for the remaining eight patients.

After completion of the SHIVC anastomosis and testing with cold perfusion, the allograft was perfused with the same solution warmed to 37°C. Further samples from the infrahepatic IVC were taken at this time. Approximately 1000 ml of perfusate was used and liver temperatures were monitored. When the liver core temperature exceeded 22°C perfusion ceased, the infrahepatic IVC was occluded and the iliac artery cannula was attached to the in-dwelling portal vein cannula used for perfusion purposes. The suprahepatic IVC clamp was removed and also the clamp on the iliac artery shunt, resulting in graft revascularization (Figure 1). If necessary, flows through the arterial shunt into the donor portal vein, measured by means of an electromagnetic flowmeter, were regulated to be less than 1000 ml per min. Haemostasis was again confirmed. The infra-hepatic IVC anastomosis was then

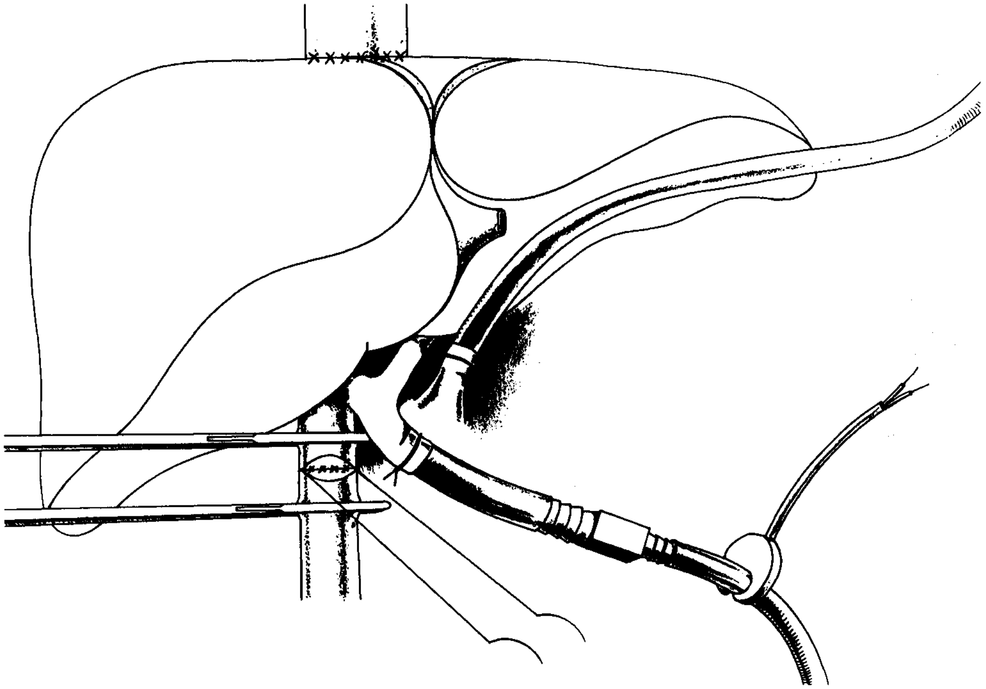

**Figure 1** The suprahepatic IVC anastomis has been completed. Revascularization follows at once by joining the iliac artery shunt to the donor portal vein perfusion catheter as shown. The portal limb of the recipient veno-venous bypass is also illustrated. The infrahepatic IVC anastomosis is being performed.

completed and released with removal of the systemic limb of the veno-venous bypass. The hepatic arterial anastomosis was then performed and released. On one occasion two donor hepatic arteries required anastomosis. If there was any doubt concerning the adequacy of arterial flow, flow measurements were made and, if the flow was unsatisfactory (less than 4 ml/kg) body weight), the anastomosis was redone. This happened on one occasion. Only when arterial flows and systemic blood pressures were satisfactory were the portal venous anastomoses performed, preceded by removal of the iliac artery shunt from the donor portal vein and the portal limb of the veno-venous bypass from the recipient portal vein. Biliary outflow was established by choledochocholedochostomy or by choledochojejunostomy. Wedge hepatic biopsies were taken before wound closure.

## RESULTS

Total ischaemic intervals for the liver allografts were 3 h 36 min – 7 h 51 min (average 5 h 10 min). Anhepatic intervals for the recipients were 55 min – 2 h 15 min (average 1 h 55 min). The blood transfusions required during operation numbered 3 – 73 units (— 30 units). With the exception of the first three patients, the arterio-portal shunts were open for 18–85 (— 46) min. Blood flows through the shunts measured 250–520 (— 352) ml/min, except on two occasions when flows of 1200 and 1500 ml/min were recorded on opening the shunts and in these cases the flows were mechanically adjusted to be less than 1000 ml/min. Pressures within the portal veins measured while the arterio-portal shunts were operating were 11–25 mmHg and were similar to the central venous pressures recorded at the same time.

At the time of revascularization there was no serious haemorrhage in any patient. There was no significant change in the heart rate in eight patients, while the heart rate increased by up to 15 beats per min in the other three. There was a fall in the mean arterial pressure at the time of revascularization from 92 ± 14 mmHg (mean ±s.d) to 83 ± 13 mmHg. Recovery of blood pressure to pre-revascularization levels had occurred usually within 5 min and always within 15 min. The cardiac index increased in 10 of the patients from a mean of 5.67 ± 1.95 $l/min/m^2$ to a mean of 7.6 ± 4.1 $l/min/m^2$. Although the cardiac index had reduced to 6.52 ± 3.36 $l/min/m^2$ within 15 min, it remained at an increased level throughout the operation, final estimates being 6.09 ± 3.33 $l/min/m^2$. Arterial blood pH values fell from 7.3 ± 0.13 units to 7.23 ± 0.12 units at the time of revascularization. Ionized calcium levels decreased from 0.9 ± 0.26 mmol/l to 0.79 ± 0.18 mmol/l. An immediate short-lived rise in serum potassium levels at the time of revascularization (mean peak value 3.7 ± 1.1 mmol/l) was followed by a sustained fall in values to below baseline levels (2.8 ± 0.6 mmol/l). Blood coagulation studies after revascularization showed a brief prolongation of the activated partial thromboplastin time (APTT) from 51 ± 27 s to 79 ± 48 s with essentially stable prothrombin index and blood platelet counts.

Biochemical analyses of samples taken of the effluent from the infrahepatic IVC at the times of cold perfusion and warm perfusion immediately before revascularization showed variations from the inflowing perfusion fluids particularly in the levels of potassium ($K^+$) and of the hepatic cellular enzymes alanine amino-transferase (ALT) and aspartate amino-transferase (AST). These levels were greatly elevated but reduced progressively during the wash-outs as shown in Table 2. Blood gas analysis and pH determinations revealed a fall in the oxygen tension in the warm

**Table 2** Levels of potassium ($K^+$), alanine amino-transferase (ALT) and aspartate amino-transferase (AST) in the hepatic allograft effluent perfusate at the geginning and end of cold and warm perfusion.

| | *Cold perfusion* | | *Warm perfusion* | |
|---|---|---|---|---|
| | *Beginning* | *End* | *Beginning* | *End* |
| $K^+$ mmol/l | 14.7–37.9 (*m* 22.3) | 11.4–29.8 (*m* 18.1) | 6.2–27.9 (*m* 11.2) | 3.8–11.7 (*m* 6.4) |
| ALT[a] units/l | 567–28000 (*m* 9078) | 118–14000 (*m* 6511) | 126–11020 (*m* 2839) | 252–2679 (*m*998) |
| AST[a] units/l | 693–25000 (*m* 8455) | 210–3378 (*m* 4306) | 256–4173 (*m* 2399) | 197–2334 (*m* 918) |

[a] Normal values for ALT and AST in our laboratory are 5–55 units/l.

perfusate from 440 ± 126 mmHg to 45 ± 26 mmHg after traversing the liver, while there was a decrease of 0.36 ± 0.12pH units.

Microscope examination of the wedge biopsy specimens taken from all livers at the end of the operations regularly revealed the presence of a thin layer of subcapsular hepatic necrosis thought to be associated with graft manipulation. Otherwise the graft cells were uniformly viable and the hepatic architecture well preserved.

In all 11 patients there was immediate evidence of function of the allografts as secretion of copious amounts of normally pigmented bile occured. All patients recovered promptly from the operation, with the exception of one who developed fitting, leading to cerebral anoxia and ultimately death. A second patient died of cerebral haemorrhage 10 days after operation. In all nine other patients evidence of continuing progressive improvement in liver function was clear. In all, haematological parameters, with the exception of platelet counts, were close to normal from the time of operation. At 24 h and 48 h postoperatively AST levels in the blood were 126–691 (*m* 274) units/l and 60–352 (*m* 160) units/l. ALT levels at these times were 152–765 (*m* 340) units/l and 83–668 (*m* 320) units/l. The levels of these enzymes and of the serum bilirubin fell continuously thereafter unless interrupted by rejection activity. Radioisotope liver scans (HIDA) performed on the day of operation or on the first or second postoperative days were always reported to show satisfactory hepatic function. Strongly pigmented bile was secreted by all patients in volumes allowing 200–400 ml per day to drain via the T-tubes. All nine (82%) remain alive and well 4–18 (*m* 11) months since transplantation, although two have impaired hepatic function because of biopsy-confirmed chronic rejection.

## DISCUSSION

The number of parameters involved in early postoperative good function in human liver allografts is so great that analysis of the role played by various factors is extremely difficult. Most parameters are non-immunological. Crucial factors include the physical state of the donor, donor liver and recipient, the pre-operative and intra-operative management of both donor and recipient together with the operative techniques employed, the fluids and methods used for preservation, and the postoperative management of the recipient.

For these reasons we are uncertain of the relative importance of the methods used here for donor hepatectomy, liver preservation and graft implantation. After experimental work in pigs that resulted in improved laboratory success rates in our hands[8] we simply translated the methds in totality to the clinical sphere. Since deficiency in any aspect of overall management usually leads to failure in liver transplantation[9] it seems likely that all the methods used by us are at least satisfactory, as all the transplanted livers functioned well.

The methods we used for donor hepatectomy were modelled on those of Starzl *et al*[10]. We do not, however, use their "pre-cooling" method of cold infusion into the liver via the splenic vein during the early dissection of the liver. Instead we prefer not to dissect the organs but to minimize operating time and the risks of donor hypotension by performing cold perfusion via vessels remote from the liver and kidneys. This technique is more akin to the "fast" donor operation described by Starzl *et al.*[11] and recently further elaborated[12]. By these methods all trauma caused by manipulations of the organs to be removed is avoided, together with any effects of arterial vasospasm that might occur as a results of dissection involving the arteries of supply to the organs.

We also chose to use oxygenated "extracellular" perfusion fluids because of success in the laboratory experiments. In both the laboratory and clinical work oxygen was taken up by the allografts during perfusion as revealed by distinct falls in the oxygen tension in the perfusion solutions after traversing the ischaemic allografts. While most groups use hyperosmolar solutions containing high levels of potassium for perfusion, in our hands a simple solution containing human serum albumin and with electrolyte concentrations similar to extracellular fluid has proved effective for preservation of all livers, the longest total ischaemic interval being 7 h and 51 min. Further, serious hyperkalaemia never occurred at revascularization, in contrast to its variable occurrence in reports from other centres. On the other hand, biochemical analysis of the effluent from the infrahepatic IVC at the times of cold and warm perfusions showed changes that reflect some organ damage. Thus, the levels of potassium were extremely high in the effluent after cold perfusion but had reduced to acceptable levels by the end of warm perfusion. Similarly, levels of ALT and AST were very high in the cold perfusate but reduced rapidly as a result of the cold and warm wash-outs.

The method of graft revascularization whereby arterial blood is shunted into the donor portal venous system allows revascularization immediately after the completion of one anastomosis (SHIVC). Without the use of the arterio-portal shunt most other groups perform the SHIVC anastomosis and then the infrahepatic IVC and portal venous anastomoses before revascularizing. This method adds to ours the time required to perform two aditional anastomoses, so increasing the total ischaemic time for the allograft and the anhepatic period for the recipient. During this additional time the graft is rewarming in the recipient, making it more vulnerable to ischaemic damage.

However, we have become wedded to our method of revascularization for a variety of other reasons. First, cannulation of the superficial femoral artery is a minor addition to preparation of a groin for veno-venous bypass, which we use regularly except in small children. Secondly, knowledge that safe liver revascularization can follow within minutes of completion of the suprahepatic IVC anastomosis encourages us to undertake meticulous haemostasis during the anhepatic interval and to perform the top anastomosis with deliberation, these steps accounting for our average anhepatic interval of 1 h 55 min. Thirdly, assured absence of haemorrhage or of significant haematological, biochemical or acid-base abnormalities at the time of revascularization is reassuring for both the surgical and the anaesthetic teams. Fourthly, a technical advantage is that the posterior aspect of the suprahepatic IVC anastomosis and the retrohepatic region are still available for inspection and suture if required after revascularization, as the liver remains freely mobile on the SHIVC anastomosis because it is not tethered by an infrahepatic anastomosis. Fifthly, the method removes the pressures of time constraints on all the anastomoses remaining after completion of the suprahepatic IVC anastomosis. Because the liver is safely revascularized, each anastomosis can be done without hurry, satisfactory blood flow can be confirmed and, if need be, anastomoses can be redone. Finally, the method provides the capability for dealing with unexpected complications that may develop during the procedure, as in two cases mentioned below.

The possibility that the method of revascularization might be damaging to the allograft was a matter of prime concern initially. However, with the flow rate from the arterial shunt into the portal vein either being less then normal portal flow when unrestricted or being adjusted to approximate normal flows, it is logical, and we have confirmed, that blood pressures within the portal system are close to normal portal pressures. Flow via the shunt is continuous and only gently pulsatile, as is the case in arterio-venous fistulas, and as evidenced by flow meter recordings and a continuous thrill palpable in the donor portal vein while the shunts were operational. With normal flows and pressures in the portal system it seems highly unlikely that haemodynamic damage could result. However, there is the further consideration that the presence of arterial concentrations of oxygen within the portal system, as opposed to the usual lower oxygen concentrations of portal blood, might be damaging. All the evidence that we can gather relating to this matter indicates that no specific hepatic cellular or other damage results from the method. Thus, animals with arterialization of the portal vein can survive for long periods with good hepatic function until the haemodynamic effects of the fistulas begin to take effect[13]; hepatic malfunction is relatively rare in the short term after arterio-portal fistulas[14]; and all haematological, biochemical and histological evidences from our own experimental work[8] showed comparatively minor damage after orthotopic liver transplantation when this method of revascularization was used. These observations are confirmed in the clinical study reported here, where the degree of graft damage as assessed by the early postoperative blood levels of liver cellular enzymes was modest, and microscopy of liver biopsy specimens taken at the end of the operations showed the preservation of normal hepatic architecture. Further, there was every indication of good early graft function virtually from the time of revascularization and continuing thereafter.

A factor that we think may have some importance is that in our method of revascularization arterial blood is provided to the ischaemic graft, whereas it is common practice to revascularize with portal venous blood. In the latter case the graft then remains without arterial blood for a further 15–20 min while the arterial anastomosis is completed. There are uncertainties in our minds as to whether revascularization with blood released from the obstructed portal circulation is always beneficial to the ischaemic allograft. It seems possible to us that in a small proportion of liver grafts, perhaps those already damaged by other factors, irrecoverable injury may occur during the time they are supported by portal venous blood alone, and that this may explain otherwise inexplicable cases of primary non-function of liver grafts. This concern is underlined by the sometimes catastrophic consequences of arterial thrombosis in the early post-transplant days, in which situation the newly implanted liver is supported with portal blood only.

If this reasoning proves correct it will be asked why it would not be more logical to shunt blood to the donor hepatic artery or revascularize after the hepatic anastomosis alone. However, these are not attractive alternatives because of the frequency of multiple hepatic arteries, the possibility of vasospasm or arterial damage resulting from the arterial manipulations, and concern should the arterial anastomosis not be perfect or should hypotension develop for any reason. If inadequacy of arterial supply occured for any reason at this stage, the consequences could be catastrophic, as after revascularization the liver graft is rewarmed and so highly susceptible to damage from ischaemia. With our method, as long as it necessary can be taken to ensure a normal arterial supply to the entire graft, and during this period the graft is perfectly perfused via the portal vein with arterial blood. Also in two cases where severe hypotension developed, once before and once after liver revascularization (because of variceal haemorrhage in the former and haemorrhage during disconnection of a porto-caval shunt in the latter), we have established that satisfactory blood flows of 200–300 ml/min pass through the arterial shunt into the low pressure portal system despite serious hypotension. It was, we believe, life-saving to be able to achieve urgent revascularization in the first case and to maintain satisfactory graft perfusion by means of the arterial shunt in both cases while these complications were dealt with.

Because of our inexperience in clinical transplantation and because we have no control series of patients, historical or concurrent, we cannot be certain whether our method of revascularization has sufficient advantages to warrant adding a further complicating manoeuvre to an already complicated procedure. Our conclusion is that the method is an alternative way to revascularize liver grafts that is safe and that may have some advantages. It has allowed us to encompass satisfactorily the initial phase of our clinical programme despite a significant proportion of high-risk patients.
